# Potential Suitable Habitat Range Shift Dynamics of the Rare Orchid *Cymbidium cyperifolium* in China Under Global Warming

**DOI:** 10.3390/plants14193084

**Published:** 2025-10-06

**Authors:** Yaqi Huang, Xiangdong Liu, Ting Chen, Chan Chen, Yibo Luo, Lu Xu, Fuxiang Cao

**Affiliations:** 1 College of Horticulture, Hunan Agricultural University, Changsha 410128, China; 2 Hunan Botanical Garden, Changsha 410116, Chinachenchan0829@163.com (C.C.); 3 Hunan Changsha-Zhuzhou-Xiangtan City Cluster National Research Station of Forest Ecosystem, Changsha 410116, China; 4 State Key Laboratory of Systematic and Evolutionary Botany, Institute of Botany, Chinese Academy of Sciences, China National Botanical Garden, Beijing 100093, China; luoyb@ibcas.ac.cn

**Keywords:** climate change, *Cymbidium cyperifolium*, habitat suitability, Maxent

## Abstract

Wild orchids, valued for their beauty and economic importance, are facing the challenges of distribution contraction and range shifts from climate change. The rare *Cymbidium cyperifolium* (class II in the List of National Key Protected Wild Plants in China, Vulnerable on the China Biodiversity Red List) remains understudied regarding its responses to climate variability. Utilizing an enhanced MaxEnt model, we predicted suitable habitats under diverse climate scenarios, revealing a potential distribution of 52.37 × 10^4^ km^2^, concentrated in eastern Yunnan, western Guangxi, the Guizhou border, and southern Hainan. *Cymbidium cyperifolium* is sensitive to climate change, and temperature annual range (Bio 7) contributes a significant 77.42% of the distribution probability (i.e., habitat suitability), highlighting temperature’s pivotal influence on its distribution. Although the overall potential distribution area and low-suitability regions in China are predicted to decrease, medium and high-suitability areas are expected to expand. The center of mass of the high-altitude habitat is concentrated in southeastern Yunnan Province, migrating just slightly, yet tending westward and northeastward. Based on these findings, we recommend the expansion of existing protected areas or the establishment of new ones for *C. cyperifolium*, particularly in eastern Yunnan and western Guangxi. Additionally, our research can serve as a reference for the ex situ conservation of *C. cyperifolium* and other orchids with similar ecological habits, underscoring the broader implications in biodiversity preservation efforts.

## 1. Introduction

Five primary factors, climate change, habitat alteration, overexploitation, pollution, and invasive alien species, are recognized as main threats to global biodiversity in the 21st century [1]. Climate change, a significant driver of global change, predominantly manifests through rising temperatures, altered precipitation patterns, and increased frequency of extreme weather events. These climatic factors influence growth, reproduction, phenology, and geographical distribution on a regional scale [2], and this impact is particularly pronounced in endemic plants [3,4]. Current biodiversity conservation strategies typically rely on the existing species distribution, but with the increasing impact of climate change, these strategies face mounting challenges. Understanding the shifts in the suitable distribution areas of species under future climate scenarios and implementing targeted conservation measures promptly is vital for enhancing biodiversity conservation effectiveness.

According to the latest Intergovernmental Panel on Climate Change (IPCC) report, global average temperature is expected to rise by 1.4 to 5.8 degrees Celsius in the 22nd century, representing an increase rate 2 to 10 times the warming rate in the 21st century. As global temperatures climb, hydrothermal conditions are anticipated to undergo unprecedented changes, leading to corresponding changes in the potential distribution of plant species [5]. The substantial rise in temperature is expected to significantly change the habitat distribution range of many species and elevate their extinction risk [6]. In recent decades, multiple studies on the relationship between species geographic distribution and global climate change have shown that over 80% of species’ geographic distribution will be affected by climate dynamics [7]. Shifts in species distribution are closely related to climate warming, and with pronounced impacts observed in high-altitude areas [8].

**Figure 1:**
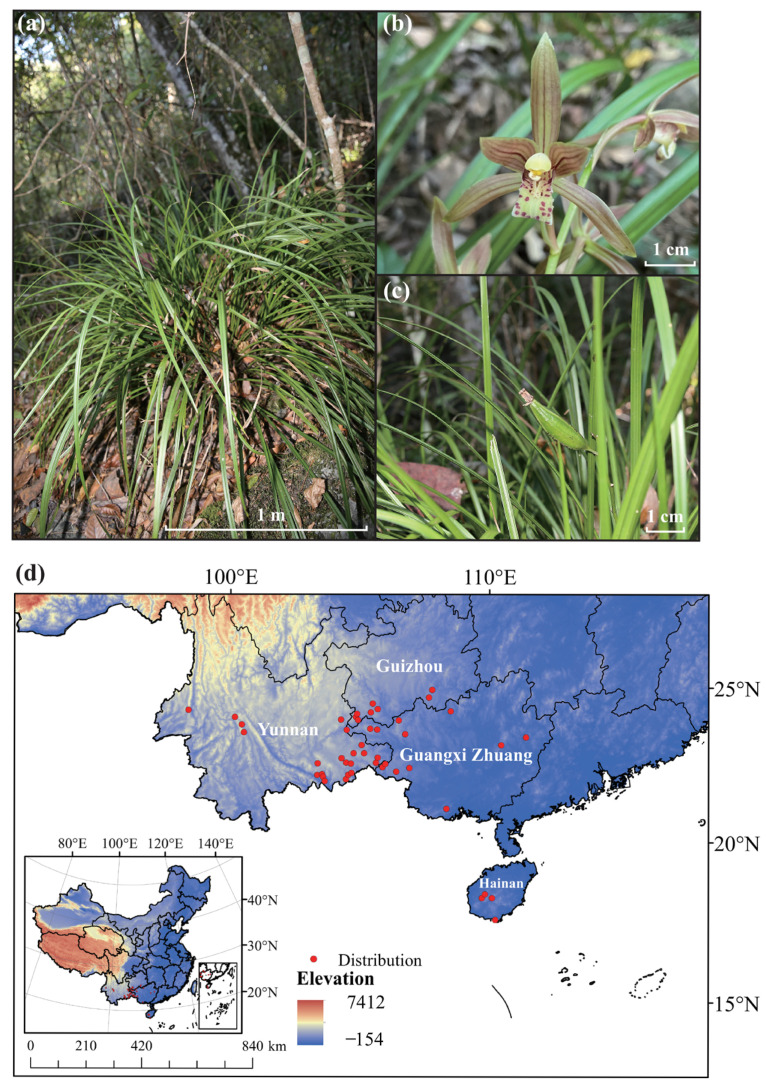
Wild characteristics and distribution of *Cymbidium cyperifolium* in China. (**a**) Wild growth of *C. cyperifolium*, (**b**) flowers of *C. cyperifolium*, (**c**) fruit of *C. cyperifolium*. (**d**) Distribution of *C. cyperifolium* in China.

The Orchidaceae family is known for its vast species diversity [9]. It is also one of the most visually appealing plant families, ranking as the second largest among angiosperms worldwide [10,11]. Within this family, the semi-epiphytic *Cymbidium cyperifolium* exhibits distinctive morphological and ecological characteristics (Figure 1a–c), and is primarily distributed in southern China, including Guangdong, Hainan, southern Guangxi, southwestern Guizhou, and southeastern Yunnan (Figure 1d). Beyond China, its range extends to Nepal, Bhutan, India, Myanmar, Thailand, Vietnam, Cambodia, and the Philippines. This perennial herb demonstrates a prolonged flowering period extending from October to February of the subsequent year. Its floral morphology produces lemon-scented flowers akin to those of *C. kanran*. The phyllotaxis of *C. cyperifolium* is particularly noteworthy, typically presenting 4–12 distichously arranged leaves that form a characteristic fan-shaped architecture, a unique trait distinguishing it from other congeneric species. Ecological investigations reveal its preference for well-drained lithophytic environments in subtropical evergreen broad-leaved forests, predominantly karst landforms at elevations between 900 and 1600 m. The Yachang Orchidaceae National Nature Reserve harbors the world’s largest known population of *C. cyperifolium*, with both population density and total abundance surpassing those of other documented localities. Population densities can reach 8.2 ± 1.3 individuals/m^2^ in optimal microhabitats, the highest documented for this species [12]. Nevertheless, there is a significant lack of documented information and scientific studies regarding the population status of *C. cyperifolium* due to habitat loss and wild collection.

Despite the vast number of orchid species, many have a limited geographical distribution range [13]. Orchids, both terrestrial and epiphytic, are particularly vulnerable to the impacts of climate change [14,15]. Currently, the long-term survival of orchids is threatened by urbanization, habitat loss and fragmentation, climate change, atmospheric nitrogen deposition, and excessive harvesting [13,16,17]. Although orchids can produce thousands of seeds in one growing season, few seeds grow into plants, and the survival rate of germinated seeds from the seedling stage to the adult stage is usually low [18,19]. Similar to other species in *Cymbidium*, the seeds of *C. cyperifolium* lack endosperm and rarely germinate under natural field conditions. Even when germination does occur, the seedling stage is highly vulnerable; minor environmental fluctuations can prevent seedlings from progressing to subsequent developmental stages [20]. Therefore, in many cases, asexual reproduction serves as a crucial mechanism for the maintenance and expansion of *C. cyperifolium* populations. Notably, 86.5% of the plants on the trade ban list released by the International Trade Organization for Endangered Species of Wild Fauna and Flora (CITES) are orchids [21]. *C. cyperifolium* is designated as a National Key Protected Wild Plants Category II in China. It is assessed as Vulnerable (VU) on the China Biodiversity Red List: Higher Plants, reflecting specific threats to its wild populations within the country [22]. Despite this, proactive conservation efforts are often insufficient to secure their long-term survival, necessitating the development of additional strategies. This study focused solely on China due to the considerable challenges of multinational research, including securing permits and complying with varying biodiversity regulations. Furthermore, species occurrence data from other countries are scarce and often unverified. This research aims to provide conservation and management recommendations at the national scale. Extending the modeling to a broader region may be possible in the future if reliable occurrence data become available. However, comprehensive studies predicting the suitable habitats for *C. cyperifolium* are currently lacking, which significantly hinders the development of effective conservation strategies.

To address the knowledge gap in the spatial distribution of the threatened *C. cyperifolium*, in this study, the Maxent model was employed to predict the potential habitat areas of *C. cyperifolium* in China under the current and future climate, thereby offering a scientific basis for its targeted conservation. Our objectives were (1) to identify the distribution of *C. cyperifolium* and understand its growth response to climate change, and (2) to forecast the potential distribution area and the migration of the center of mass of high-suitability zones under climate change in China. This study will provide a scientific basis for the long-term planning and cultivation of *C. cyperifolium* in China, offering theoretical support for the relocation and in situ conservation of *C. cyperifolium* and other orchids with similar ecological requirements. This strategy aims not only to protect and expand the population of *C. cyperifolium* but also to conserve its entire associated biological community. This need arises from the species’ specific habitat requirements and complex ecological relationships, such as those with specialized pollinators and mycorrhizal fungi [23], as well as the vulnerability of its habitat to degradation, including severe rocky desertification in karst environments and limited capacity for autonomous recovery [24,25]. By creating ecological corridors to link fragmented populations and securing vital habitats, this approach efficiently conserves a mutually dependent ecosystem, thus transforming a single-species conservation initiative into a comprehensive ecosystem preservation strategy.

## 2. Results

### 2.1. Model Parameter Optimization

For parameter selection, the regularization multiplier (RM) is set to 0.5, and the feature class (FC) is set to LQ; the Akaike Information Criterion corrected (AICc) value is minimized (delta AICc = 0) (Table 1). This indicates that the model’s complexity is the lowest under these parameter settings, and consequently, the degree of overfitting is relatively low. Hence, the study selected RM = 0.5 and FC = LQ as the optimal parameters for the final model setting.

### 2.2. Prediction of Potential Suitable Areas for Current C. cyperifolium

#### 2.2.1. Model Fitting Results

**Figure 2:**
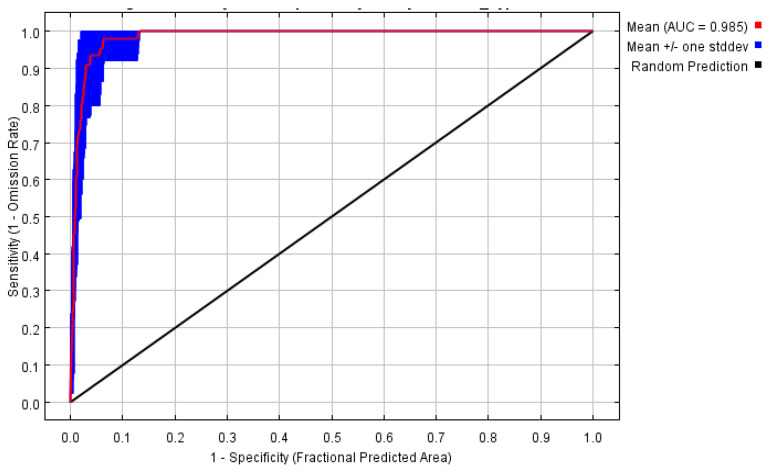
Maxent ROC curve and AUC value.

This section evaluates the fitting accuracy of the Maxent model. Under current conditions, the Receiver Operating Characteristic (ROC) curve demonstrates an Area Under the Curve (AUC) value of 0.985, which significantly exceeds the random prediction baseline of 0.5 (Figure 2). This suggests that the Maxent model is both stable and reliable, and it can be employed for accurately predicting the distribution of *C. cyperifolium* in suitable areas across China.

#### 2.2.2. Distribution of Suitable Habitats in the Current Context

**Figure 3:**
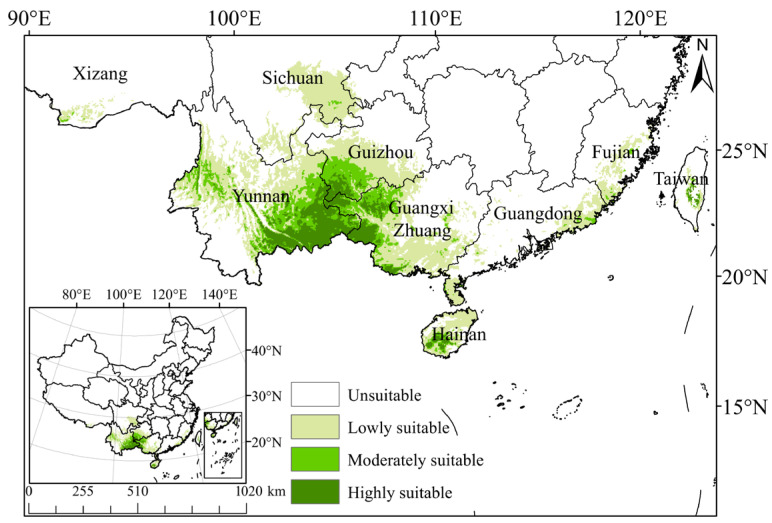
Potential suitable habitats of *C. cyperifolium* in China predicted by Maxent model.

The Maxent model predicts that the total suitable growth area of *C. cyperifolium* spans 52.37 × 10^4^ km^2^, comprising 5.45% of the entire study area (Table 2). Areas of high suitability are predominantly located in the southeastern Yunnan Province, southwestern Guizhou Province, the western Guangxi Zhuang Autonomous Region, and southwestern Hainan Province, covering 7.67 × 10^4^ km^2^ and accounting for 0.80% of the entire research area. Moderate suitability regions are primarily situated in the southwest subtropical regions in China, including Yunnan, Guangxi Zhuang Autonomous Region, Guizhou, Hainan, etc. In contrast to the highly suitable areas, the moderately suitable area also extends into the eastern Guangdong Province and central Fujian Province, covering 10.12 × 10^4^ km^2^ or 1.05% of the total study area. The poorly suitable area encompasses the majority of Yunnan Province, eastern and southern Guangxi Province, eastern Guizhou Province, southeastern Sichuan Province, and some parts of Guangdong Province and Fujian Province in Taiwan Province, with an area of 34.58 × 10^4^ km^2^, representing 3.60% of the entire study area (Figure 3).

### 2.3. Meteorological Factor Response Curve

**Figure 4:**
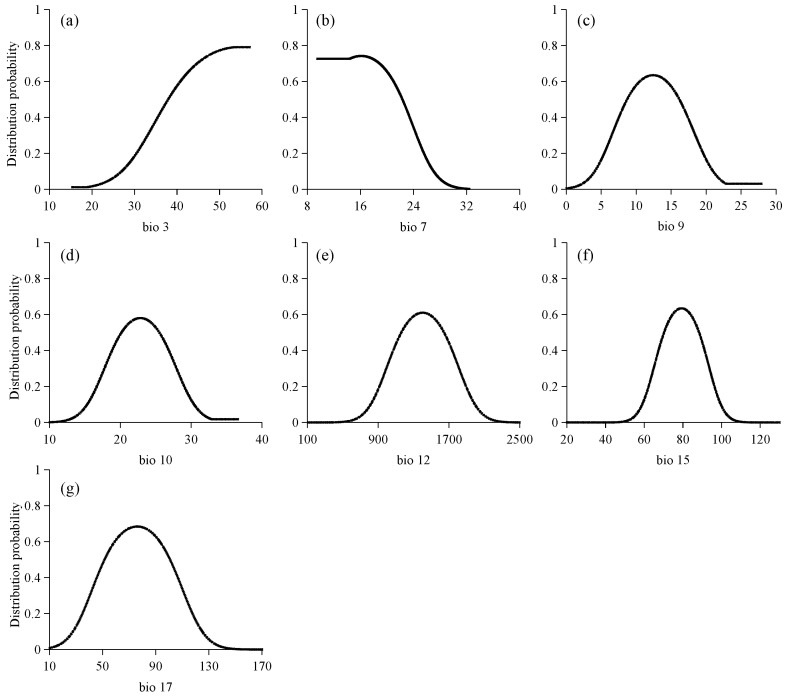
Response curve for 7 climate variables: (**a**) isothermality, (**b**) temperature annual range, (**c**) mean temperature of driest quarter, (**d**) mean temperature of warmest quarter, (**e**) annual precipitation, (**f**) precipitation seasonality, (**g**) precipitation of driest quarter.

It is generally accepted that when the probability of occurrence exceeds 0.5, the corresponding environmental factor is deemed suitable for species distribution. Figure 4 illustrates the response curves for seven climate factors influencing the distribution of potential suitable areas for *C. cyperifolium*. Most climate variable response curves exhibit an approximately normal distribution pattern, where the probability of occurrence initially increases and then decreases as climate conditions change.

When considering isotherm (Bio 3), the existence probability of *C. cyperifolium* increases with rising isotherm values and surpasses 0.5 when the isotherm exceeds 24.07. Conversely, the probability of occurrence decreases with the expansion of the annual temperature average range (Bio 7), specifically, when this range exceeds 22.33 °C, it indicates unsuitability for *C. cyperifolium* growth. These trends suggest the species is sensitive to meteorological variations. Mean temperature of driest quarter (Bio 9) spans 8.74 °C to 16.17 °C, while the mean temperature of warmest quarter (Bio 10) ranges from 20.09 °C to 25.51 °C. Annual precipitation (Bio 12) varies between 1157 mm and 1644 mm, and the precipitation seasonality (Bio 15) ranges from 70.26 to 88.38. Additionally, the precipitation of driest quarter (Bio 17) ranges between 51.55 mm and 100.66 mm.

**Figure 5:**
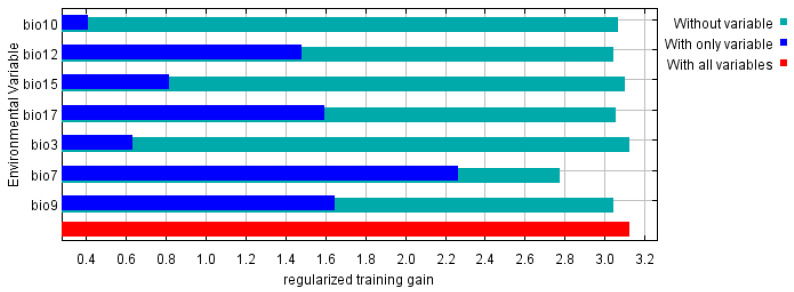
Jacknife text of environment variables in MaxEnt.

The Jackknife test results for each climate variable indicate that when simulations are conducted using a single variable, the three variables that contribute the most to the model’s regularization training gains are the temperature annual range (Bio 7), the mean temperature of driest quarter (Bio 9), and the precipitation of driest quarter (Bio 17) (Figure 5). These climate variables provide more comprehensive and effective information than others. The temperature annual range (Bio 7), with contributions of 77.42%, contributes significantly to the model’s performance. This suggests that both the direct and indirect impacts on the distribution range of *C. cyperifolium* as critical limiting or promoting factors.

### 2.4. Changes in Potential Distribution Areas Under Future Climate Change

#### 2.4.1. Distribution Range of Potential Suitable Areas for *C. cyperifolium* Under Future Climate Scenarios

**Figure 6:**
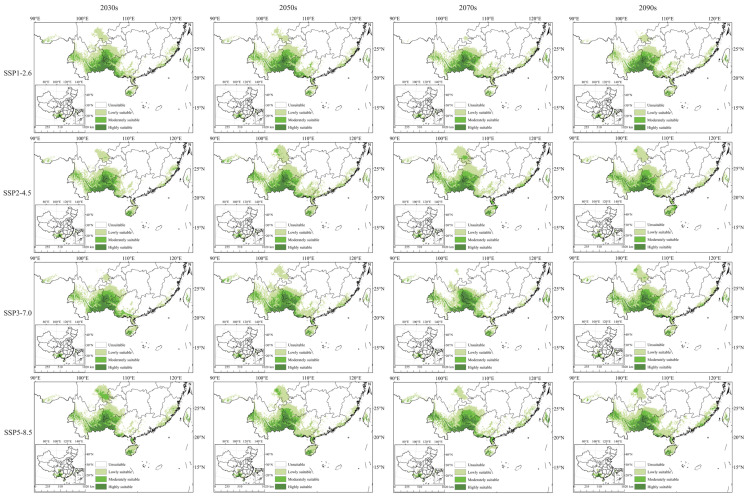
Potential suitable habitats for *C. cyperifolium* in different climate scenarios.

Figure 6 indicates that the geographic distribution of potential suitable areas for *C. cyperifolium* under the four future climate scenarios remains largely consistent with the current scenario. These continue to be concentrated in the subtropical climate regions of China, with highly suitable areas mainly located in the southeastern Yunnan Province, the southwestern Guizhou Province, the western Guangxi Zhuang Autonomous Prefecture, the southwestern of Hainan Province, and the central Taiwan Province. Significant changes in area primarily occur in the southern region of Sichuan. The optimal area during the 2070s SSP1–2.6 and SSP3–7.0 periods is relatively small, whereas SSP2–4.5 and SSP5–8.5 scenarios display larger areas.

The total area of suitable habitat for *C. cyperifolium* fluctuates over time, peaking in the SSP2–4.5 scenario circa 2070s, with a 13.44% increase from the current scenario. Conversely, the SSP3–7.0 scenario of the 2070s shows the smallest total suitable habitat area, decreasing by 19% from the current scenario, though its high-suitability area is 87,100 km^2^, a 13.56% increase over the current scenario.

Overall, with the increase in time, the high-altitude habitat of *C. cyperifolium* exhibits only a slight decline in the SSP2–4.5 scenario during the 2030s and 2090s, decreasing by 6.5% and 7.3%, respectively. All other periods and scenarios show an increasing trend, suggesting that while *C. cyperifolium* growth is sensitive to future climate change, these changes will not threaten the species’ survival in the short term.

#### 2.4.2. Potential Habitat Area of *C. cyperifolium* Under Future Climate Scenarios

**Figure 7:**
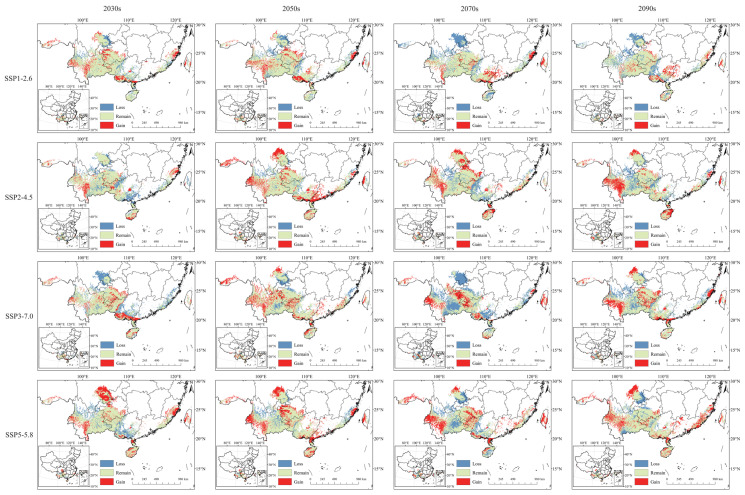
Change in potential suitable habitats of *C. cyperifolium*. The red part in the figure represents the area where the fitness increases, the blue part represents the area where the fitness decreases, and the green part represents the area where the fitness remains.

This process generated a schematic representation of the potential geographic distribution changes for *C. cyperifolium* under projected climate conditions compared to current environments (Figure 7).

Compared with the current climate scenario, the changes in the suitable habitat area of *C. cyperifolium* are inconsistent in different periods and situations. Notably, suitable habitat regions are increasing toward southwestern Yunnan, southeastern Guangdong Province, and central southern Hainan Province, while contracting in central eastern Yunnan Province. Across China, there is a trend of suitable habitat areas gradually spreading and expanding towards the periphery of concentrated distribution zones. Overall, the distribution of *C. cyperifolium* is gradually shifting towards high-altitude areas.

### 2.5. Centroid Migration of Suitable Area in the Future Climate Scenarios

**Figure 8:**
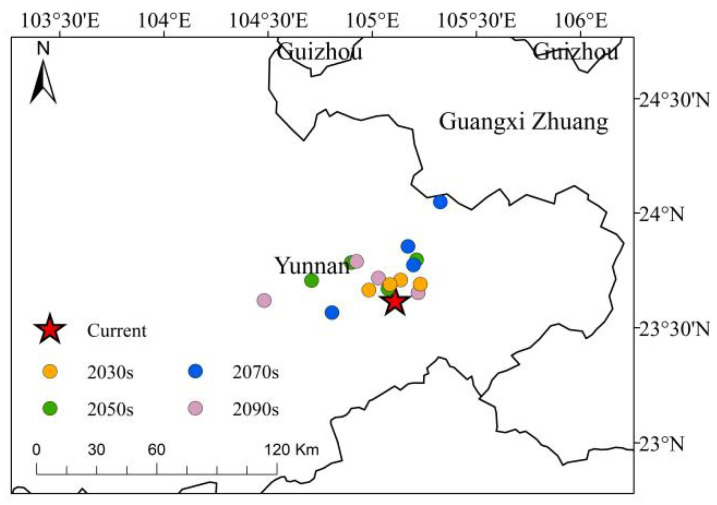
Distribution of centroids of *C. cyperifolium* in highly suitable habitats under different climatic scenarios. Red pentagram represent current; yellow represents 2030s; green represents 2050s; blue represents 2070s; purple represents 2090s.

Under various climate scenarios, the centroid of the high-suitability area for *C. cyperifolium* is concentrated in the southeastern region of Yunnan Province. Compared to the current situation, this centroid predominantly shifts westward and northward, albeit over relatively short distance (Figure 8). The average migration distances across four different periods are as follows: 0.11 km in the 2030s period, 0.23 km in the 2050s period, 0.30 km in the 2070s period, and 0.28 km in the 2090s period. Studies indicate that the centroid’s degree of dispersion increases over time across different contexts. From 2021 to 2040, under the SSP1–2.6 scenario, the centroid migration in high-altitude areas is minimal (0.09 km) compared to the current scenario. Conversely, in the 2090s under the SSP2–4.5 scenario, the migration of the centroid for high-altitude habitats is the most pronounced at 0.62 km. Although the center of *C. cyperifolium* high-altitude habitat shifts varying distances under different climate scenarios, no consistent directional pattern of movement has been identified between the movement distance and climate scenarios across different periods.

## 3. Discussion

### 3.1. Distribution of C. cyperifolium in China

We found that the potential suitable areas of *C. cyperifolium* are concentrated in the present day, widely distributed across the coastal regions of Guangdong, Guangxi, and Taiwan, as well as in southeast of Xizang, Yunnan, southwest Sichuan, and central to southwest of Guizhou, aligning with existing records. Currently, *C. cyperifolium* in China is mainly distributed in Guangxi, Yunnan, Hainan, and Guizhou regions. The world’s largest cluster of *C. cyperifolium* was discovered in Leye County, northwest Guangxi Zhuang Autonomous Region (106°11′31”~106°27′04” E, 24°44′16”~24°53′58 N), characterized by a typical subtropical monsoon climate [26]. This suggests that the microenvironment climate and geographical features of this region are highly suitable for the growth of *C. cyperifolium*.

The geographical distribution of plants is the result of long-term interactions with climate. Changes in plant distribution patterns result from shifts in climate factors within suitable areas, as well as the species’ distribution characteristics and adaptability to extreme climatic conditions [27]. However, the distribution area of *C. cyperifolium* in China is relatively small at only 5.45%, which is less than that of most endangered Orchidaceae species such as *Cypripedium macranthos* [28], *C. japonicum* [29], and *C. shanxiense* [30]. This indicates a strong environmental dependence. Species with broader ecological niches usually exhibit greater adaptability and resource utilization ability [31], suggesting that *C. cyperifolium* is more affected by environmental changes, and therefore deserves more attention regarding trends in its potential habitat.

### 3.2. Limiting Factors of C. cyperifolium

*C. cyperifolium* is extremely sensitive to climate conditions, particularly temperature changes (Figure 5). Key temperature metrics, including isothermality (Bio3), temperature annual range (Bio 7), mean temperature of driest quarter (Bio9), mean temperature of warmest quarter (Bio 10), and three precipitation indicators—annual precipitation (Bio 12), precipitation seasonality (Bio 15), and precipitation of driest quarter (Bio 17)—are the primary climatic factors influencing this species’ potential distribution in China. Similar to many species, climate warming is the major driver of shifts in distribution patterns [32,33]. Importantly, the contribution rate of Bio 7 reached 77.42%, underscoring the crucial role of temperature in determining the distribution of *C. cyperifolium*.

We have identified the annual temperature range (Bio7) as the primary climatic factor determining the macro-scale distribution of *C. cyperifolium* in China (Figure 5). Nonetheless, the model paradoxically categorizes some known occurrence points within this range as having low climatic suitability. This discrepancy underscores the multi-scale nature of species distribution patterns. At the macro scale, climate factors set the climatic boundaries for the species’ potential range; at the local scale, particularly for Orchidaceae, species persist in these seemingly unsuitable grids by taking advantage of more favorable microenvironments (such as sheltered ravines or forest understories [23,34]) or relying on biotic factors like mycorrhizal fungi [35,36] and pollinators [37]. These populations may function as ecological sink populations, relying on dispersal from source areas to maintain their presence or continue to exist at the edge of their physiological tolerance. As a result, the species’ overall suitable habitat does not appear to be significantly affected under future scenarios (Figure 6 and Figure 7). These findings not only confirm the dominant role of climate factors but also highlight the critical importance of identifying microrefugia—small-scale environmental sanctuaries that can offer temporary refuge to species amidst macro-scale climate changes—and marginal habitats, which are essential for developing effective conservation strategies and accurately assessing the species’ vulnerability to environmental changes.

This sensitivity is related to the growth characteristics of the spider plant. *C. cyperifolium* typically grows in shaded forest areas with minimal light. However, due to the relatively low annual precipitation in the distribution area, the dry and wet seasons are obvious [38]. Due to the predominance of gravel and thin soil layers, as well as poor water retention, these plants often exist in a semi-epiphytic state [39]. The morphology and anatomical structure of the leaves and roots of *C. cyperifolium* show adaptations typical of both wet and dry environments, including thin leaves, raised stomata, and a high shoot-to-root ratio typical of wet habitats, as well as a thick cuticle, well-developed mechanical tissues, compact cells, crystal idioblasts, fleshy roots with velamen, and thickened endodermal and exodermal walls, characteristic of dry environments, indicating a high degree of adaptability to environmental conditions [39]. The variable thickness of leaf veins increases the surface area, enhancing photosynthesis and leaf rigidity. Internally, frequent fiber cell clusters within the mesophyll, along with well-developed vessels and fibers in the vascular bundles, provide mechanical support, facilitating leaf extension and efficient light use under forest canopies [40]. These structural features at the microscopic level explain the temperature sensitivity of *C. cyperifolium*.

### 3.3. Changes in Potential Habitable Areas

Global warming may lead to the redistribution of precipitation in different regions, thereby altering plant distribution patterns [41]. Research indicates that climate warming tends to cause most species to migrate towards higher latitudes and altitudes [8,42,43]. Our findings align with this, showing that the suitable habitat of *C. cyperifolium* gradually expands from the border of Yunnan and Guangxi to the surrounding regions, migrating to higher altitudes such as Yunnan and southeastern Tibet, possibly due to shifts in climate zones in Yunnan Province (Figure 6).

In most instances, the centroid migrates westward and northward (Figure 8). The temperature increase in high-altitude regions is more pronounced compared to lower altitudes, significantly altering species’ geographical distributions [44], encouraging species to move northward or migrate to higher altitudes [45,46]. Due to the combined influence of the Qinghai–Tibet Plateau, the East Asian monsoon, and Southwest monsoon, climate change in the karst region of central Yunnan, situated on a low-latitude plateau, is particularly pronounced and complex, making it sensitive to climate change [47]. Cheng et al. [48] noted that climate zones in Yunnan are shifting, with an increase in subtropical areas and a decrease in northern subtropical and temperate regions, demonstrating a northward movement and a trend of expansion into high-altitude areas.

Variability in *C. cyperifolium*’s potential habitat at different times and contexts is notable, with the expansion range being most significant during the SSP2–4.5 scenario in the 2050s. This stems from a less extreme land use and aerosol pathway, offering a milder warming trend [49]. *C. cyperifolium*, a warmth- and humidity-loving plant, will likely see increased suitable habitats due to climate warming, as these conditions favor light and temperature-preferring species. Conversely, global warming is predicted to increase rainfall in mid-to-high-latitude regions, while drought events may become more frequent in low-latitude areas [50], pressuring species like *C. cyperifolium* to migrate to higher latitudes for more favorable growth conditions [51]. Additionally, increasing SSPs may negatively impact orchid distribution [52], as seen with a decrease in suitable habitat areas of *C. cyperifolium* in the SSP3–7.0 scenario, a phenomenon similar to observations with *Horsfieldia hainanensis* Merr [53] reminding us to monitor potential adverse effects of climate change on species in the future.

To ensure the long-term survival of *C. cyperifolium*, we recommend prioritizing the identified key suitability areas, especially those with high predicted probabilities (e.g., in Yunnan and Guangxi), for establishing formal protected areas. However, recognizing the logistical and financial challenges of creating new state-managed reserves, a complementary strategy is vital. For suitable habitats within community forests or on private lands, conservation goals can be effectively advanced by empowering local stakeholders. This involves supporting Indigenous and Community Conserved Areas (ICCAs) and promoting mechanisms such as conservation easements, which provide legal and economic incentives for habitat protection without relying solely on government administration. Integrating formal protection with collaborative governance can establish a resilient conservation network for *C. cyperifolium* across the landscape [54,55].

## 4. Materials and Methods

### 4.1. Species Distribution Location

Distribution data for *C. cyperifolium* in China were acquired from several sources (1) a field investigations conducted in 2022 October at the National Nature Reserve of Orchidaceae Plants in Baise City, Guangxi Province (106°11′31″–106°27′04″ E, 24°44′16″–24°53′58″ W), (2) the Global Biodiversity Information Facility (GBIF, https://www.gbif.org/, accessed on 8 March 2023) [56] (3) the Chinese Virtual Herbarium (CVH, http://www.cvh.ac.cn/, accessed on 23 September 2024), and (4) the published literature. All occurrence records obtained from GBIF and CVH were rigorously validated for taxonomic identity and spatial accuracy through expert verification, coordinate screening, and removal of duplicates. To reduce spatial autocorrelation, distribution points were thinned to retain only those spaced at least 2.5 km apart. This refinement yielded 46 geographical distribution records of *C. cyperifolium* (Figure 1d).

### 4.2. Climate Data Sourcing and Screening

#### 4.2.1. Climate Data Sourcing

The climate variables used in this study were sourced from the World Climate Database (http://www.worldclim.org/, with a resolution of 2.5 arc minutes). Nineteen bioclimatic factors were obtained (Table 3), with significant impacts on species distribution across five periods—the current period (1950–2000), 2030s (2020–2040), 2050s (2040–2060), 2070s (2060–2080), and 2090s (2080–2100)—with a resolution of 2.5 min. We selected the BCC-CSM2-MR climate system model, and employed four Shared Socioeconomic Pathways (SSPs) scenarios in this model, to describe the quantitative relationship between the extent of climate change and socio-economic development paths [52]. These scenarios include SSP1–2.6 (low-carbon and sustainable development pathway), SSP2–4.5 (intermediate emissions and middle-of-the-road pathway), SSP3–7.0 (high emissions and regional rivalry pathway), and SSP5–8.5 (very high emissions and fossil-fueled development pathway), each representing varying greenhouse gas emission concentration scenarios from low to high.

#### 4.2.2. Climate Factors Screening

The correlation between climate variables can result in the overfitting of model predictions. To mitigate this risk, Pearson correlation analysis was conducted on 19 layers of Bioclimatic variables using the band set statistical tool in ArcGIS 10.7 software (version 10.7.0.10450; Esri, Redlands, CA, USA). Concurrently, climate factors were screened based on their contribution to the distribution of *C. cyperifolium*. Following this analysis, and considering seven climate variables were ultimately selected, including four temperature indicators such as isothermality (Bio 3), temperature annual range (Bio 7), mean temperature of driest quarter (Bio 9), mean temperature of warmest quarter (bio 10), and three precipitation indicators, such as annual precipitation (Bio 12), precipitation seasonality (Bio 15), and precipitation of driest quarter (Bio 17). Based on the longitude and latitude of the existing distribution records of *C. cyperifolium*, the climatic data under the current scenario was extracted using ArcGIS 10.7, and the results are presented in Table 4.

### 4.3. Species Distribution Predictive Modeling Framework

#### 4.3.1. Maxent Model

The Maxent (Maximum Entropy) model has been extensively utilized to predict geographic distributions across various species and is acknowledged as one of the highest-performing and most widely used species distribution models [57]. Studies have frequently compared the performance of various niche models, with most results demonstrating that Maxent outperforms other modeling approaches [58,59]. Phillips et al. [60] developed Maxent software (https://biodiversityinformatics.amnh.org/open_source/maxent/, accessed on 8 January 2024) in 2004, a Java-based application for predicting potential geographic distributions of species. In this study, we employed the Maxent model, Version 3.4.1 (https://biodiversityinformatics.amnh.org/open_source/maxent/, accessed on 8 January 2024) for model construction.

#### 4.3.2. Implementation of MaxEnt Modeling Workflow

The filtered distribution point data for *C. cyperifolium* was saved in Excel in the format “species name, longitude, latitude” and exported as a CSV file necessary for running Maxent software. Regarding environmental variable data, the “Grid to ASCII” tool in ArcGIS 10.7 is utilized to convert all environmental variables into ASC files compatible with Maxent software (Version 3.4.1). Subsequently, *C. cyperifolium* distribution point data and climate variable layer are loaded in the appropriate format. The maximum number of background points is set to 10,000, and a cross-validation method is employed to extract test samples by partitioning all known distribution data into ten subsets—one for testing and nine for training—to enhance data utilization. This process was repeated ten times, with the final ASCII output file representing the average of these repetitions.

#### 4.3.3. Model Parameterization and Tuning

When utilizing the Maxent model to predict potential species distribution areas, although most researchers employ default parameters, some have highlighted that models built with these defaults can be overly complex, potentially leading to overfitting and challenges in result interpretation. To address this issue, Muscarella et al. [61] developed the program package ENMeval in R (4.2.1) to optimize the model parameters. This package adjusts two parameters of the model, Regulation Multiplier (RM) and Feature Combination (FC). It assesses model complexity using various parameter combinations and identifies those that reduce complexity, thereby optimizing the model.

In this study, the ENMeval package was employed to optimize the model using the block method, which partitioned the 46 distribution records of *C. cyperifolium* distribution records into four approximately equal parts, with three parts designated for training and one for testing. The RM varied from 0.5 to 4.0, in increments of 0.5. For FC parameters, the Maxent model provides five types of features, linear (L), quadratic (Q), fragmented (Hint, H), product (P), and threshold (T). Six feature combinations were used for testing, L, LQ, H, LQH, LQHP, and LQHPT. The ENMeval package evaluated 48 parameter combinations, selecting the one that minimized the delta AICc to establish the optimal model.

#### 4.3.4. Model Verification

The Area Under the Curve (AUC) value was chosen as the metric for assessing the accuracy of the model prediction in this study. The Maxent model software can generate the Receiver Operating Characteristic (ROC) curve corresponding to its prediction results and calculate its AUC value.

#### 4.3.5. Response Curves

The Maxent model generates response curves that elucidate the impact of major environmental factors on species distribution. These curves allow for the determination of the relationship between the probability of species occurrence and environmental variables.

### 4.4. Prediction of Potential Suitable Habitats for C. cyperifolium

#### 4.4.1. Prediction of Suitable Habitats

Utilizing species distribution points alongside seven chosen environmental factors within the Maxent modeling framework, this study anticipated the potential distribution of *C. cyperifolium* across China under current climatic conditions. ArcGIS (version 10.7) facilitated the processing the prediction results and subsequent reclassification. The study area was categorized into four suitability classes based on the fitness index, unsuitable (0–0.1), poorly suitable (0.1–0.3), moderately suitable (0.3–0.5), and highly suitable (0.5–1).

#### 4.4.2. Migration of Centroids in High Suitable Habitats Areas

To further analyze changes in the potential habitat areas of *C. cyperifolium* across varying climate scenarios, ArcGIS 10.7 was employed to spatially overlay distribution maps of potential habitat areas under diverse future climate scenarios with current climate distribution maps. Additionally, the centroid calculation tool in ArcGIS 10.7 was used to derive the centroids of the high-altitude areas of *C. cyperifolium* across different climate scenarios. This analysis aimed to explore potential migration trends of suitable habitats for *C. cyperifolium* in the context of climate change.

## 5. Conclusions

In this study, we employed the MaxEnt model toassess the suitable habitat conditions for *C. cyperifolium* under varying climatic scenarios. The findings indicate that *C. cyperifolium* is predominantly distributed in eastern Yunnan, western Guangxi Zhuang Autonomous Region, at the border of Guizhou Province, and southern Hainan Island. Its suitable growth area spans approximately 52.37 × 10^4^ km^2^, constituting 5.45% of the total study area. *C. cyperifolium* demonstrates sensitivity to climate fluctuations, with the center of mass of its high-potential distribution area migrating towards the west and northeast. We propose expanding expansion or establishment protected areas to safeguard this orchid’s habitats, especially in eastern Yunnan and western Guangxi, offering a model for conserving similarly ecological orchids. This study provides essential data for devising protective strategies and offers a reference for the ex situ conservation of *C. cyperifolium* and orchids with similar ecological characteristics.

## Figures and Tables

**Table 1:** Evaluation results of Maxent model under different parameter combinations.

RM	FC	AICc ^6^	Delta. AICc
0.5	H ^1^	2821.38	1907.19
	L ^2^	975.66	61.46
	LQ ^3^	914.19	0.00
	LQH	NA	NA
	LQHP ^4^	NA	NA
	LQHPT ^5^	NA	NA
1	H	1595.29	681.09
	L	978.67	64.48
	LQ	930.89	16.70
	LQH	1107.90	193.70
	LQHP	1061.47	147.28
	LQHPT	1095.81	181.61
1.5	H	1371.42	457.23
	L	982.82	68.63
	LQ	931.11	16.91
	LQH	1017.23	103.03
	LQHP	1020.07	105.88
	LQHPT	1011.44	97.25
2	H	1070.73	156.54
	L	984.86	70.66
	LQ	938.16	23.97
	LQH	1016.05	101.86
	LQHP	969.75	55.56
	LQHPT	1014.11	99.91
2.5	H	1009.80	95.61
	L	986.81	72.62
	LQ	937.75	23.56
	LQH	947.89	33.69
	LQHP	945.78	31.59
	LQHPT	947.13	32.93
3	H	1069.49	155.30
	L	989.13	74.94
	LQ	941.79	27.60
	LQH	925.86	11.66
	LQHP	952.43	38.24
	LQHPT	940.73	26.54
3.5	H	1007.02	92.83
	L	991.79	77.60
	LQ	945.93	31.74
	LQH	931.43	17.24
	LQHP	945.19	30.99
	LQHPT	959.40	45.21
4	H	999.54	85.35
	L	994.68	80.48
	LQ	950.18	35.99
	LQH	941.31	27.12
	LQHP	952.50	38.31
	LQHPT	956.92	42.73

^1^ Hint (H), ^2^ linear (L), ^3^ quadratic (Q), ^4^ product (P), ^5^ threshold (T), ^6^ AICc represent Akaike’s lnformation Criterion with Corrected Akaike’s Information Criterion.

**Table 2:** Areas of suitable habitats of *C. cyperifolium* under climate scenarios and their proportion of the national area/10^4^ km^2^.

Time	Shared Socio-Economic Pathways SSPs	All Suitable		Poorly Suitable		Moderately Suitable		Highly Suitable	
		Area	%	Area	%	Area	%	Area	%
Current		52.37	5.45	34.58	3.60	10.12	1.05	7.67	0.80
2030s	1–2.6	53.75	5.59	34.90	3.63	10.88	1.13	7.97	0.83
	2–4.5	48.42	5.03	30.76	3.20	10.49	1.09	7.17	0.75
	3–7.0	46.03	4.79	26.22	2.73	11.45	1.19	8.36	0.87
	5–8.5	55.06	5.72	34.24	3.56	12.86	1.34	7.96	0.83
2050s	1–2.6	51.84	5.39	32.09	3.34	11.77	1.22	7.98	0.83
	2–4.5	59.41	6.18	38.08	3.96	13.10	1.36	8.23	0.86
	3–7.0	55.17	5.74	34.01	3.54	12.68	1.32	8.48	0.88
	5–8.5	58.44	6.08	37.27	3.88	12.37	1.29	8.80	0.91
2070s	1–2.6	46.15	4.80	28.49	2.96	9.93	1.03	7.73	0.80
	2–4.5	54.53	5.67	35.00	3.64	12.07	1.25	7.46	0.78
	3–7.0	42.42	4.41	24.80	2.58	8.91	0.93	8.71	0.91
	5–8.5	52.59	5.47	31.28	3.25	12.85	1.34	8.46	0.88
2090s	1–2.6	47.04	4.89	29.70	3.09	9.64	1.00	7.70	0.80
	2–4.5	53.60	5.57	34.50	3.59	11.99	1.25	7.11	0.74
	3–7.0	52.64	5.47	31.96	3.32	11.72	1.22	8.96	0.93
	5–8.5	57.42	5.97	36.40	3.78	12.84	1.34	8.18	0.85

**Table 3:** Environmental variables used for modeling the potentially suitable habitat of *C. cyperifolium*.

Code	Name of Bioclimate Variables	Unit
Bio 1	Annual Mean Temperature	℃
Bio 2	Mean Diurnal Range (Mean of monthly)	℃
Bio 3	Isothermality	%
Bio 4	Temperature Seasonality	℃
Bio 5	Max Temperature of Warmest Month	℃
Bio 6	Min Temperature of Coldest Month	℃
Bio 7	Temperature Annual Range	℃
Bio 8	Mean Temperature of Wettest Quarter	℃
Bio 9	Mean Temperature of Driest Quarter	℃
Bio 10	Mean Temperature of Warmest Quarter	℃
Bio 11	Mean Temperature of Coldest Quarter	℃
Bio 12	Annual Precipitation	mm
Bio 13	Precipitation of Wettest Month	mm
Bio 14	Precipitation of Driest Month	mm
Bio 15	Precipitation Seasonality	mm
Bio 16	Precipitation of Wettest Quarter	mm
Bio 17	Precipitation of Driest Quarter	mm
Bio 18	Precipitation of Warmest Quarter	mm
Bio 19	Precipitation of Coldest Quarter	mm

**Table 4:** Climatic conditions for the growth of *C. cyperifolium* under the current scenario.

Bio 3 %	Bio 7 ℃	Bio 9 ℃	Bio 10 ℃	Bio 12 mm	Bio 15 mm	Bio 17 mm
47.93	22.06	8.82	19.32	1263	76.16	65
48.78	22.68	9.13	19.72	921	76.71	52
48.66	23.36	12.60	23.25	960	81.90	49
48.38	21.70	11.31	19.95	1029	80.01	54
42.99	19.30	12.96	23.50	1396	81.60	74
47.80	20.69	8.84	18.86	1300	86.03	51
42.37	19.09	10.22	20.84	1555	81.38	78
41.49	19.16	10.25	21.08	1630	81.24	80
39.99	18.74	11.10	21.02	1762	83.44	80
43.40	23.34	9.05	21.64	1108	79.80	62
44.50	21.28	9.76	21.16	1297	86.04	48
42.07	20.69	9.87	21.45	1356	86.96	52
41.42	19.92	10.01	21.24	1667	95.96	52
40.57	22.53	13.37	24.23	1133	80.13	62
40.92	20.19	11.80	23.26	1561	92.21	53
41.62	20.68	9.44	21.05	1392	87.90	53
41.11	20.52	11.09	22.71	1530	91.96	53
42.36	21.46	8.82	20.68	1244	83.15	53
39.49	22.59	8.17	21.04	1235	80.10	65
38.31	23.26	9.52	22.98	1257	80.61	67
37.90	22.67	9.64	22.87	1240	80.47	65
42.22	22.96	10.27	22.87	1076	79.47	45
40.48	21.55	9.81	21.94	1205	83.87	45
38.12	22.77	9.09	22.32	1257	84.34	56
36.55	23.54	8.08	22.05	1309	80.52	65
34.70	23.59	7.95	22.35	1307	79.62	64
38.70	21.07	11.04	23.36	1367	87.15	54
36.72	22.58	9.31	22.73	1268	87.41	52
39.45	21.52	12.02	24.45	1291	87.49	45
33.68	23.46	8.68	23.11	1320	81.40	62
37.20	20.70	11.60	24.06	1420	86.86	58
36.38	20.60	10.54	23.02	1416	88.00	58
34.81	20.46	12.49	25.23	1501	87.90	74
34.04	23.44	8.66	22.99	1249	82.00	52
33.13	23.00	9.71	24.07	1305	81.85	63
33.34	21.43	14.82	26.97	1466	83.53	87
30.48	26.44	6.63	23.51	1274	68.50	83
28.73	25.89	4.90	21.82	1293	66.73	89
30.46	19.84	13.40	24.59	1862	82.00	105
29.49	26.06	9.04	25.91	1409	67.39	114
42.60	15.74	17.50	25.65	1420	79.03	52
42.46	15.90	15.65	23.89	1594	77.59	64
43.19	15.72	16.80	24.83	1591	76.18	70
48.10	14.41	22.85	28.75	1456	77.74	76
29.57	23.83	11.25	24.75	1613	65.65	132
29.83	25.13	12.32	26.47	1553	66.11	129

## Data Availability

The original contributions presented in this study are included in the article. Further inquiries can be directed to the corresponding authors.
